# Effect of nutritional therapy in Emery–Dreifuss muscular dystrophy: a case report

**DOI:** 10.3389/fnut.2024.1343548

**Published:** 2024-04-29

**Authors:** Filippo Valoriani, Giovanni Pinelli, Silvia Gabriele, Renata Menozzi

**Affiliations:** ^1^Division of Metabolic Diseases and Clinical Nutrition, Department of Specialist Medicines, University Hospital of Modena and Reggio Emilia - Policlinico, Modena, Italy; ^2^Internal and Emergency Medicine, Modena - Ospedale Civile Baggiovara, Azienda Ospedaliero-Universitaria Modena, Modena, Italy

**Keywords:** Emery–Dreifuss muscular dystrophy, EDMD, muscular dystrophies, total parenteral nutrition, TPN, resting energy expenditure, REE

## Abstract

Emery–Dreifuss muscular dystrophy (EDMD) is a rare, inherited human disease. Similar to other neuromuscular dystrophies, EDMD is clinically characterized by muscle atrophy and weakness, multi-joint contractures with spine rigidity, and cardiomyopathy. Over time, muscular weakness can lead to dysphagia and a severe lowering of body mass index (BMI), worsening the prognosis. We present the case of a young male patient affected by EDMD, admitted to the hospital for pneumothorax in a severe state of undernourishment. The patient was treated with total parenteral nutrition (TPN) with Smofkabiven®, supplemented with micronutrients (vitamins and trace elements), and with minimal enteral nutrition through food. Within a year, the patient gained 8.5 kg and kept his body weight stable for the 6 years of the follow-up. In this study, we show that TPN ensures the nutritional requirements of EDMD patients in a safe and well-tolerated manner, allowing a considerable and stable improvement in nutritional status, which has a positive impact on the disease itself and the patients’ quality of life.

## Introduction

EDMD is a rare slowly progressive muscular dystrophy. It is a genetically heterogeneous neuromuscular orphan spectrum disease affecting approximately 0.3–0.4 in 100,000 people (1). Several genes have been implicated in the pathogenesis of EDMD, such as EMD, LMNA, SYNE1, SYNE2, FHL1, and TMEM43 (2); however, there are still more causative genes yet to be discovered, as over 60% of patients do not have mutations in EMD or LMNA, the most common genes involved in EDMD pathogenesis (2). In EDMD, the onset of symptoms occurs within the first decade of life. Contractures of the elbows, neck extensor muscles, and Achilles’ tendons appear to be the first symptoms of the disease and occur before muscle weakness and deterioration (3). Progressive muscle degeneration begins at the end of the second decade of life in a humeroperoneal distribution (3). The cervical spine rigidity may become prominent enough to alter neck anatomy and impair swallowing, which is already present due to muscle weakness (3). Cardiac complications are found in the majority of EDMD patients, the onset is during the teenage years, and they worsen over time, frequently leading to sudden death (4). Nutritional complications often arise in EDMD dystrophies, but they are sometimes underestimated. Progressive muscle weakness leads to chewing, swallowing, and digestive problems, resulting in weight loss, which further worsens muscular performance (5). A negative energy balance due to unmet nutritional requirements, caused by an insufficient protein energy intake, may worsen the clinical outcome (6). Resting energy expenditure (REE) is mostly spent by lean body mass (LBM), composed of muscle mass and viscera, the latter contributing 10 times more to the REE (7). Only one study has explored the correlation between body composition and REE in patients with Emery–Dreifuss muscular dystrophy (EDMD). These changes include elevated body fat mass and a noteworthy decrease in LBM. Despite these alterations, EDMD patients demonstrate an increased REE, particularly when the REE is adjusted for LBM (8). These findings are unexpected because prominent progression of muscular dystrophy with no change in the viscera should cause a decrease in the daily REE. Patients with EDMD do not lose significant LBM, and muscle mass does not decrease remarkably. Therefore, causes other than muscle wasting may have been involved in these REE abnormalities, and perhaps metabolic alterations and respiratory distress may play a role (8). This evidence is in line with what has been previously observed in patients with Duchenne muscular dystrophy (DMD) (9, 10).

We present the case of an adult male patient affected by EDMD who was treated for 6 years with TPN in combination with minimal enteral feeding. We describe the positive effects of artificial nutritional therapy on nutritional parameters, body weight, and physical performance in this particular clinical setting.

## Case report

A 26-year-old male patient affected by EDMD (laminopathy type A/C, LMNA gene, NM_170707, c.523_537del (p.Ala175_Ala179del)) was admitted to the hospital in July 2015 for an acute respiratory failure caused by a bilateral spontaneous pneumothorax drained with a CT-guided catheter positioning. The patient had been admitted due to worsening respiratory symptoms, including desaturation at home, requiring continuous non-invasive mechanical ventilation (NIMV) therapy in the prone position during the day and a supine position at night. In addition to EDMD with complete dysautonomia but without cognitive deterioration, the patient had a history of chronic atrial fibrillation treated with Coumadin and a previous right pneumothorax (2011), which was surgically treated. Upon admission, the patient presented with dyspnea but was alert, oriented, and cooperative. The physical examination revealed generalized loss of subcutaneous adipose tissue and concurrent compromise of muscle mass (muscle wasting in the temporal, deltoid, and quadriceps muscles) without dependent edema. He presented a flat and manageable abdomen with no ascites. Moreover, he had dry skin and mucous membranes indicative of dehydration. The ECG showed a non-sinus rhythm consistent with atrial fibrillation. Radiological investigations revealed bilateral spontaneous pneumothorax, which was treated with guided pigtail TAC drainage, resulting in the gradual improvement of hypoxemic respiratory status. A follow-up CT scan showed minimal persistence of bilateral pneumothorax (approximately 1 cm thickness). Since there were no ventilatory repercussions, their oxygen saturation values were stable, and they did not experience any fatigue or pain, it was decided not to proceed with further drainage maneuvers. During hospitalization, the patient did not develop fever and received a blood transfusion for transient anemia. Upon discharge, the patient was in good general condition but still required continuous NIV, supplemented with oxygen during nighttime hours.

During the first nutritional assessment at hospital admission, we registered the following parameters: weight 22.5 kg, height 1.64 m, and body mass index (BMI) 8.36 kg/m^2^. The patient weighed 33 kg until he was 10 years old, and then he had a significant weight loss. Until January 2014, his usual body mass was 23.8 kg. In June 2015, body weight was 22.8 kg and decreased to 22.5 kg a month later; therefore, in 12 months, the patient had an involuntary weight loss of 5.4%, compared to usual body weight. The patient presented dysphagia to solids, leading to a progressive decrease in oral intake, especially in the 6 months prior to hospitalization. This impacted the average daily oral energy intake, reducing it down to 500–600 kcal/day, corresponding to 50% of his nutrient requirement. The oral water intake was approximately 150–200 mL/day.

During hospitalization, the already low oral nutrition was completely suspended due to NIV. Due to severe malnutrition, the patient was treated with artificial nutrition. In particular, a midline catheter was inserted into the patient’s superior vena cava, to act as a peripherally inserted central catheter (PICC). The patient received a TPN regimen with 986 mL/day of Smofkabiven® (=900 kcal non-protein +50 g amino acids) to be administered in 24 h, daily with micronutrients (vitamins and trace elements). TPN was gradually implemented to reduce the risk of refeeding syndrome, as recommended by the American Society for Parental and Enteral Nutrition (ASPEN) guidelines (11). Furthermore, the patient was nourished with minimal enteral nutrition with natural foods (less than 200 kcal/day). A high calorie, high protein oral was also prescribed, but never actually assumed. NIV was continued during the hospitalization. After a month, the patient was discharged from the hospital with a clear improvement in his general clinical conditions, even though he needed to remain on continuous NIV. The patient was sent home with the following nutritional therapy: (1) TPN with Smofkabiven®, to be administered within approximately 18 h (overnight), along with daily micronutrients as recommended by international guidelines; (2) minimal enteral feeding using natural foods administered orally. Continuous NIV was prescribed, with nasal olive during the daytime and with a mask overnight. In addition, to start home enteral nutrition, as suggested by the European Society for Clinical Nutrition and Metabolism (ESPEN) guidelines, we scheduled the placement of a percutaneous endoscopic gastrostomy (PEG) tube, which was to be performed a month later during the nutritional follow-up (12). However, when the patient came back for control, he expressed the desire not to have the PEG tube, to avoid the constant need to adopt the prone position. Following the patient’s wish, despite the potential endoscopic feasibility of PEG placement, we decided not to insert the PEG tube but continued TPN with Smofkabiven®, along with micronutrients as optimized the month before. TPN was associated with minimal enteral feeding with natural foods as desired by the patient: 200–250 kcal/day +5–10 g protein/day and 150–200 mL/day of water.

The patient underwent clinical, metabolic, and nutritional monitoring, undertaken routinely during the 6 years of the TPN at home (Table 1). No electrolyte or fluid alterations (i.e., related to metabolic toxicity) were observed. The patient kept lipid levels and blood-chemical parameters of hepatic (cytolysis and cholestasis) and renal functions within the normal range. Any signs of pressure injury, aspiration pneumonia, or edema were detected. Patients did not develop heart failure during the follow-up period. It is also important to note that there were no infectious episodes related to venous access. The PICC has been carefully maintained; in the 6 years of treatment, it has been replaced twice, once following its suspected obstruction and the second time after its accidental removal during the dressing practice.

**Table 1:** Hematological and biochemical indices measured during the quarterly clinical-nutritional monitoring of home parenteral nutrition (HPN) are described within the entire duration of the treatment.

		2015			2016			2017			2018			2019			2020			2021		
Parameter	Unit of measurement	Min	Max	Mean	Min	Max	Mean	Min	Max	Mean	Min	Max	Mean	Min	Max	Mean	Min	Max	Mean	Min	Max	Mean
Creatinine	mg/dl	0.4	1.1	**0.7**	0.5	1	**0.8**	0.9	1.1	**0.8**	0.8	1	**0.7**	0.9	1.2	**0.8**	0.9	1.1	**0.9**	0.9	1.3	**1**
AST	U/L	2	27	**12**	12	20	**11**	13	27	**18**	12	32	**20**	20	37	**21**	18	36	**27**	17	35	**25**
ALT	U/L	7	32	**24**	10	28	**22**	18	37	**26**	22	30	**29**	18	37	**24**	28	39	**30**	30	35	**31**
Gamma GT	U/L	22	44	**32**	12	40	**16**	20	35	**22**	24	44	**26**	12	36	**31**	14	40	**23**	13	49	**29**
Blood sugar level	mg/dl	90	110	**80**	86	105	**95**	88	103	**95**	93	104	**101**	90	112	**106**	95	110	**109**	100	105	**101**
Transferrin	mg/dl	89	100	**90**	106	150	**124**	192	250	**203**	190	240	**210**	220	263	**258**	203	230	**225**	140	220	**190**
Pre-albumin	mg/dl	6	19	**13**	20	32	**25**	28	35	**30**	27	40	**33**	29	45	**37**	18	35	**25**	16	22	**18**
C-Reactive Protein CRP	mg/dl	1.5	1	**1.1**	0.5	1	**0.5**	0.6	1.1	**0.7**	0.5	0.9	**0.7**	0.5	0.9	**0.8**	0.8	1.5	**1.1**	0.5	1.3	**1.1**
Triglycerid	mg/dl	93	117	**112**	98	136	**125**	100	130	**118**	104	125	**110**	99	143	**126**	110	155	**134**	120	145	**138**
Total Cholesterol	mg/dl	75	104	**82**	83	115	**92**	95	118	**104**	99	125	**119**	120	174	**156**	168	195	**173**	148	163	**152**
Sodium	mEq/l	133	145	**138**	136	142	**140**	135	145	**139**	134	144	**141**	136	147	**141**	137	145	**140**	135	144	**138**
Potassium	mEq/l	3.3	4.9	**3.6**	3.5	4.8	**3.9**	3.7	5	**4.2**	3.8	5.1	**4.4**	4	5.3	**5**	3.9	4.9	**4.8**	3.4	4.9	**4.4**
Phosphorus	mg/dl	1.9	3.4	**2.2**	2.5	4.4	**3.2**	2.6	4.6	**3.2**	2.4	4.8	**3.3**	2.9	4.9	**4**	3	3.9	**3.6**	2.5	3.9	**2.9**

Bold values represent the average values in the patient.

**Figure 1:**
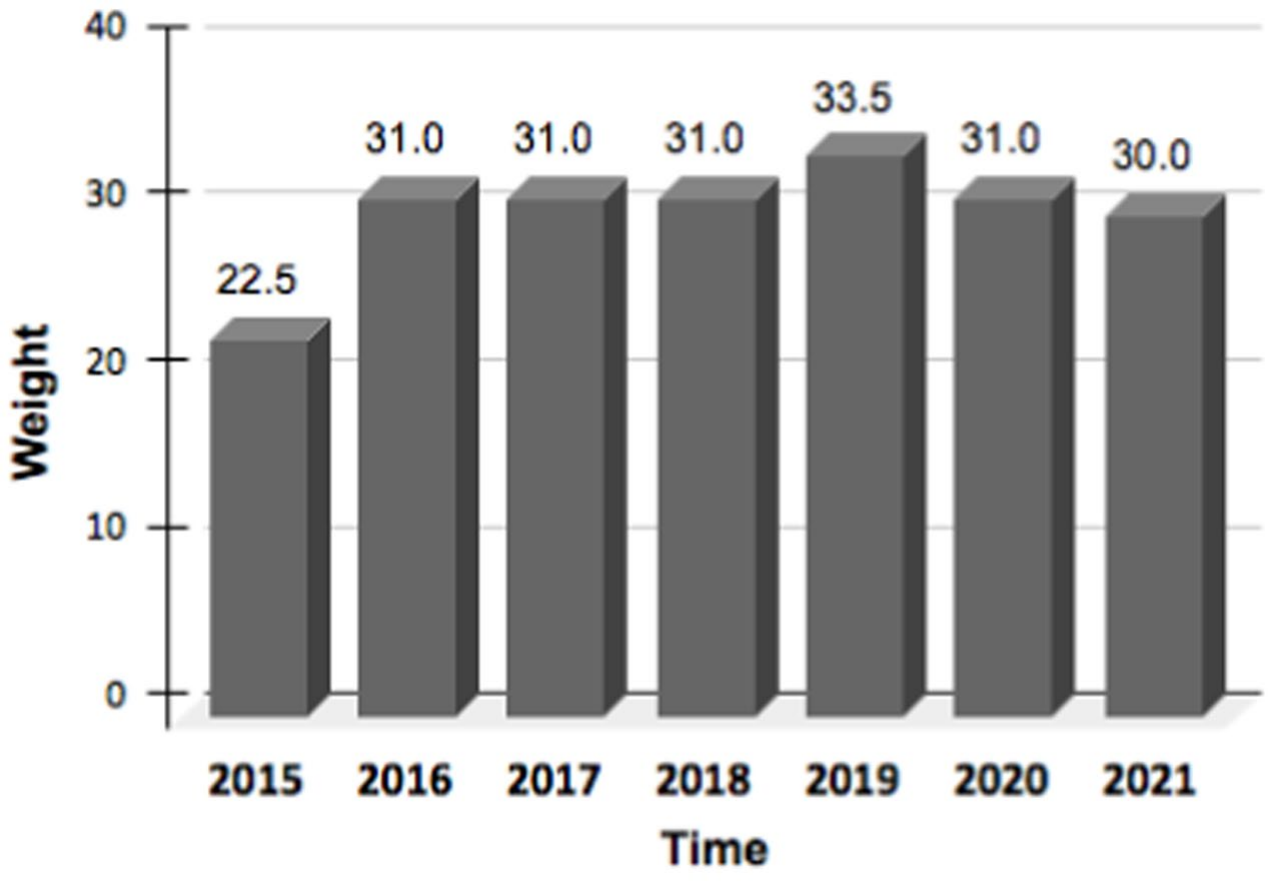
Patient body weight during the whole follow-up period, starting from 2015 to 2021.

The patient’s body weight improved and was maintained during the 6 years of treatment (Figure 1). While assessments of the quality of life using validated instruments were not conducted, the patient reported a noticeable and widespread improvement in subjective wellbeing and energy perception associated with weight recovery (patient perspective). Furthermore, the nutritional solution was consistently perceived as comfortable without negatively impacting the ability to carry out the daily activities that the patient was able and willing to engage in.

## Discussion

Life expectancy has increased in patients with muscular dystrophies due to medical interventions such as home ventilation, physical therapy, pacemaker or defibrillator placement, ACE inhibitors, and artificial nutrition (3). In the absence of therapeutic solutions for EDMD and other muscular dystrophies, long-term management of patients consists of appropriate clinical monitoring and symptomatic treatment (13). Nutrition could be considered a key parameter to monitor because excessive weight loss can worsen the overall physical condition of the patient (5). However, further studies are needed to better understand the effect of nutritional therapy on clinical outcomes in EDMD.

We presented the case of a patient affected by EDMD with a severe malnutrition status and a low BMI. It means that the patient was treated with Total Parenteral Nutrition (TPN), in synergy with minimal enteral feeding, for 6 years; we show that this type of nutritional therapy has contributed, over time, to ensure, in a safe and well-tolerated manner, the patient daily nutritional requirements. The patient gained weight within a year and kept it stable during the 6 years of follow-up. In this rare clinical context, TPN has made possible a significant improvement in the patient’s health and life expectancy. In addition, the patient had consistently reported a good overall tolerance to the home parenteral nutrition (HPN) treatment and an improvement in subjective well-being associated with weight recovery.

Although it was not possible to implement total enteral nutrition as recommended (12) due to the patient’s refusal, total parenteral nutrition (TPN) ensured adequate and satisfactory nutritional status. Additionally, no clinical or metabolic complications arose, as demonstrated by the trend in hematological and biochemical parameters (blood glucose, lipid profile, and hepato-renal function) throughout the entire duration of the treatment (Table 1).

EDMD typically results from a structural or functional defect in one or more proteins comprising the nuclear envelope. A deficiency or a mutation affecting any of these proteins can result in loss of structural integrity of the nucleus, which can be particularly problematic for tissues that are frequently under stress, including cardiac and skeletal muscles (2). Muscle deterioration and neck anatomy alterations lead to dysphagia over time, compromising calorie intake and resulting in severe weight loss (3). EDMD patients have an increased body fat mass and a significant decrease in LBM (8). Despite the decrease in muscle mass with no prominent change in the viscera, which accounts for the majority of REE, EDMD patients have an increased REE (7, 8). These observations suggest that an increased level of energy consumption, due to either heat consumption or metabolic changes, may require greater caloric intake than expected to maintain body weight. These alterations in REE, if not counterbalanced by increased caloric intake, may contribute to weight loss and a further deterioration in muscle performance.

Currently, there is a lack of data regarding the effects of nutritional therapy in EDMD, and no specific guidelines have been published. This report may suggest the feasibility and safety of nutritional therapy as an indication for improving the overall conditions of affected patients.

## Conclusion

When enteral nutrition is not achievable, TPN can be a feasible and safe nutritional therapy for patients affected by EDMD, allowing for sufficient protein and calorie intake. This approach helps mitigate signs of malnourishment and improves overall conditions with good patient tolerance.

## Data availability statement

The raw data supporting the conclusions of this article will be made available by the authors, without undue reservation.

## Ethics statement

Ethical approval was not required for the study involving human samples in accordance with the local legislation and institutional requirements. Written informed consent for participation in this study was provided by the participants’ legal guardians/next of kin. Written informed consent was obtained from the individual(s) for the publication of any potentially identifiable images or data included in this article.

## Author contributions

FV: Conceptualization, Investigation, Writing – original draft. GP: Conceptualization, Investigation, Writing – review & editing. SG: Investigation, Writing – review & editing. RM: Conceptualization, Investigation, Writing – review & editing.
